# Rewiring mTOR signaling in Alzheimer’s disease: emerging mTOR modulators beyond oncology

**DOI:** 10.1042/BSR20260203

**Published:** 2026-06-10

**Authors:** Benedikt Jakob Lohnes, Aneta Myskova, Arti Tyagi, Udo Frank Hartwig, Nitesh Kumar Poddar

**Affiliations:** 1 Department of Hematology and Medical Oncology, University Medical Center of the Johannes Gutenberg-University Mainz, 55131 Mainz, Germany; 2 Biochemistry & Molecular Biology Laboratory, Department of Biosciences, Manipal University Jaipur, Jaipur-Ajmer Express Highway, Dehmi Kalan, Near GVK Toll Plaza, Jaipur, Rajasthan 303007, India; 3 Department of Analytical Chemistry, University of Chemistry & Technology Prague, Prague, Czech Republic; 4 Life Sciences Division, Getinge Netherlands, Heertjeslaan 2, 2629 JG Delft, Netherlands; 5 Research Center for Immunotherapy (FZI), University Medical Center, Johannes Gutenberg-University, Mainz, 55131 Mainz, Germany; 6 Center for Health and Therapeutic Research (CHTR), Manipal University Jaipur, Jaipur-Ajmer Express Highway, Dehmi Kalan, Near GVK Toll Plaza, Jaipur, Rajasthan 303007, India

**Keywords:** Alzheimers disease, Cancer, inhibitors, mechanistic target of rapamycin, neurodegeneration, PI3K/AKT pathway

## Abstract

While Alzheimer’s disease (AD) is the most common cause of dementia, curative treatments remain unavailable. Despite distinct pathologies between AD and cancer, shared dysregulation of the PI3K-AKT-mTOR signaling pathway promotes both disease states. mTOR activity significantly contributes to AD hallmarks, including amyloid-beta production, tau hyperphosphorylation, and altered metabolism and autophagy through mTOR-mediated signaling and downstream targets such as BACE-1, GSK-3β, and AChE. Consequently, mTOR-modulating compounds, demonstrating promising results in oncology, present a viable strategy to potentially halt or reverse AD progression. This review discusses the potential application of 37 mTOR pathway-modulating compounds, many originally developed for cancer treatment, given their shared molecular targets. We systematically classified the compounds based on their origin as marine, plant-derived, structural analogs, and synthetic compounds. This framework reveals a fundamental trade-off, as the structural novelty and pleiotropic effects of natural products are often counterbalanced by poor pharmacokinetics, whereas the pharmacological precision of synthetic compounds is frequently limited by compensatory feedback loops. Furthermore, we analyze translational challenges, including balancing efficacy with toxicity, limitations in blood-brain barrier penetration, and the need for patient stratification using robust biomarkers. We conclude that the most promising therapeutic approach for AD involves synergistically combining natural products with rational synthetic design. Leveraging natural products as a source of novel chemical scaffolds and employing targeted synthetic engineering to overcome their pharmacokinetic limitations, this strategy moves beyond blunt pathway inhibition. Ultimately, this enables a highly nuanced modulation of the mTOR network, providing the basis for future preclinical and clinical drug development in AD.

## Introduction

The mammalian target of rapamycin (mTOR) functions as a central regulator of cell growth, proliferation, metabolism, and survival, as well as in multiple age-related pathologies, including cancer, type 2 diabetes, and neurodegenerative disorders such as Alzheimer’s disease (AD) [1]. mTOR is a serine/threonine kinase belonging to the phosphatidylinositol 3-kinase-related kinase (PIKK) family and functions within two distinct complexes, mTORC1 and mTORC2. In addition to shared subunits like mTOR, mLST8, and Deptor, mTOR1 consists of Raptor and PRAS40 [2,3] and regulates anabolic processes like protein synthesis, lipid metabolism, and ribosome biogenesis [4] while showing a high sensitivity to rapamycin [5,6]. In contrast, mTORC2 is additionally composed of Rictor, Sin1, and Protor [2,3], is insensitive to rapamycin, and is involved in cell proliferation and cytoskeletal organization [1,3].

While the inhibition of the mTOR pathway using rapamycin and its analogs (rapalogs) has demonstrated therapeutic potential in various cancers [7], the use in neurodegenerative diseases remains largely experimental. However, due to pathological mechanisms such as protein aggregation or impaired autophagy, targeting mTOR signaling represents a promising strategy for neurodegenerative conditions, which enhances autophagic clearance of misfolded proteins, reduces neuroinflammation, and improves neuronal survival, thereby highlighting the need for further research.

### AD pathology

AD is the leading cause of dementia worldwide [8], characterized by progressive impairments in memory, cognition, and mental health [9,10] as well as life-threatening complications [11]. While the symptoms exceed the typical effects of aging, no curative treatments are available [12]. Additionally, metabolic syndrome (MetS), encompassing obesity, dyslipidemia, insulin resistance, arterial hypertension, and glucose intolerance, not only shares several risk factors with AD but also contributes to its development [13,14]. These conditions are strongly associated with dysfunction of the mTOR signaling pathway, suggesting that mTOR dysregulation may play a central role in the pathogenesis of metabolic and neurodegenerative diseases.

The pathogenesis of AD is primarily driven by the accumulation of amyloid-beta (Aβ) plaques and neurofibrillary tangles (NFTs), composed of hyperphosphorylated tau protein. Aberrant enzymatic cleavage of the amyloid precursor protein (APP) by β- and γ-secretases results in Aβ fragments, which aggregate into oligomers, fibrils, and plaques. These aggregates disrupt neuronal function by causing synaptic loss, axonal damage, and impaired neurotransmission [15,16]. Similarly, hyperphosphorylation of tau protein promotes NFT formation, leading to microtubule dissociation and instability, further compromising neuronal health [17]. Furthermore, it is proposed that this hyperphosphorylation is strongly influenced by Aβ deposits and mTOR hyperactivation, thereby promoting the formation of paired helical filaments (PHFs) and NFTs [18], ultimately leading to instability-related disorders referred to as tauopathies [19].

### Shared mTOR dysregulation in cancer and AD

The pathology of AD is profoundly influenced by cellular and metabolic disruptions, including mitochondrial dysfunction, oxidative stress, neuroinflammation, and dysregulations of glucose or lipid metabolism [20]. Aberrant mTOR activation not only promotes the aggregation and accumulation of Aβ [21] and tau hyperphosphorylation [22] but also contributes to neuroinflammation and impairs autophagic clearance [23]. Consequently, targeting mTOR and its extended regulatory network, including the PI3K/AKT, AMPK, and GSK-3β pathways, represents a broad and promising therapeutic landscape.

Interestingly, dysregulation of the PI3K/AKT/mTOR axis is a well-established driver of oncogenesis, where hyperactivation leads to uncontrolled cell proliferation, metabolic reprogramming, and increased survival [24]. Given that both pathologies involve dysregulated PI3K-AKT-mTOR signaling, albeit in distinct cellular contexts and with differing downstream consequences, there is increasing interest in repurposing mTOR-targeted therapies beyond oncology, leveraging context-dependent regulatory mechanisms, and understanding the divergent outcomes of its dysregulation in proliferative versus degenerative disease states.

### The PI3K-AKT-TSC-mTOR signaling axis

Under physiological conditions, the PI3K/AKT pathway is involved in regulating cell growth, cell cycle progression, proliferation, metabolism, survival, oxidative stress, and autophagy [25].

**Figure 1:**
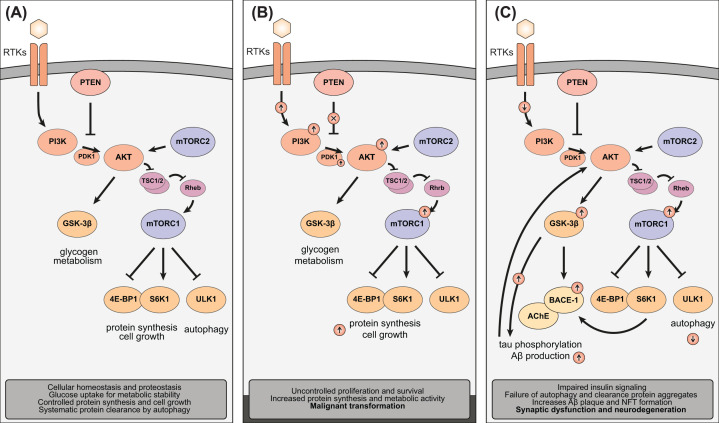
Schematic overview of the mTOR regulatory network The regulatory network influencing mTOR-related signaling is illustrated under (**A**) physiological conditions, (**B**) during cancer promotion, and (**C**) AD pathology, highlighting the key molecular contributors to signaling and biological functions.

The binding of growth factors such as insulin to receptor tyrosine kinases (RTKs) activates PI3K, generating phosphatidylinositol (3,4,5)-trisphosphate (PIP3). This recruits 3-phosphoinositide-dependent kinase-1 (PDK1) to the cell membrane, leading to AKT phosphorylation and activation [26], with AKT signaling further modulated by the antagonistic tumor suppressor PTEN [27] or the mTORC2 complex [28,29]. Downstream, AKT regulates cellular functions through two main branches. First, AKT phosphorylates and inactivates the tuberous sclerosis complex (TSC1/TSC2). This inactivation relieves the inhibitory function of TSC2 toward the small GTPase Rheb (Ras homolog enriched in brain), directly activating mTORC1 in its active GTP-bound state [30]. Once activated, mTORC1 promotes protein synthesis, lipid biosynthesis, and mitochondrial function via the ribosomal protein S6 kinase (S6K1) and eukaryotic initiation factor 4E-binding protein 1 (4E-BP1), while inhibiting autophagy via targets such as ULK1 [31,32]. Second, AKT directly phosphorylates and inhibits glycogen synthase kinase-3β (GSK-3β) as a key regulator of glycogen metabolism [33] (Figure 1A).

In numerous cancers, constitutive activation of this tightly controlled pathway, driven by RTK overexpression, activating PI3K mutations, or loss of PTEN function, promotes uncontrolled cell proliferation, metabolic reprogramming, and suppression of cell death pathways like apoptosis and autophagy [27,31,32], by hyperactivation of AKT, mTORC1, and mTORC2 (Figure 1B). In contrast, the dysregulation in AD is more complex and presents a central paradox, as mTORC1 signaling remains pathologically hyperactive even though upstream insulin signaling is often impaired. The persistent activation is driven by alternative stimuli characteristic of AD pathology, including direct stimulation by Aβ oligomers and pro-inflammatory cytokines such as TNF-α and IL-1β, which are abundant during chronic neuroinflammation [23,34]. The aberrant mTORC1 activity disrupts neuronal proteostasis, promoting protein synthesis through its canonical targets S6K1 and 4E-BP1 [31], thereby increasing the translation of pathogenic proteins such as BACE-1 and further impairing neuronal insulin sensitivity [35]. Simultaneously, mTORC1 suppresses neuroprotective autophagy by phosphorylating and inhibiting the ULK1 complex, thereby reducing the clearance of Aβ and tau aggregates [32,36]. Furthermore, dysregulated AKT signaling leads to insufficient inhibition of GSK-3β, resulting in the hyperphosphorylation of tau, which further contributes to microtubule instability, neurodegeneration, and synaptic dysfunction [37–39] (Figure 1C). Ultimately, while oncology relies on inhibition of this pathway, the distinct, paradoxical hyperactivation in AD highlights a critical research gap of the need for modulators that selectively dampen aberrant mTORC1 activity without further impairing neuronal insulin sensitivity.

### Targeting downstream mediators: BACE-1 and AChE

As pivotal enzymes in AD pathology, β-site amyloid precursor protein-cleaving enzyme 1 (BACE-1) and acetylcholinesterase (AChE) are tightly linked to the mTOR network. While BACE-1 initiates the production of Aβ peptides by cleavage of APP, elevated levels in AD patients are associated with increased amyloidogenesis and disease progression [40], with BACE-1 expression highly dysregulated by both hyperactive mTORC1 and hyperactive GSK-3β [19,41].

Similarly, acetylcholinesterase (AChE) is associated with the pathology beyond its primary role in hydrolyzing acetylcholine in cholinergic neurotransmission. By stabilizing the aggregation of Aβ peptides at the synaptic cleft, AChE promotes the formation of amyloid plaques and NFTs [42]. Moreover, the soluble Aβ oligomers further act as a chronic stimulus that feeds back to pathologically activate the mTOR signaling pathway, linking cholinergic dysfunction to the central mTOR-mediated cellular dysregulation and neurotoxicity [43–45].

## Classification of mTOR modulators

**Figure 2:**
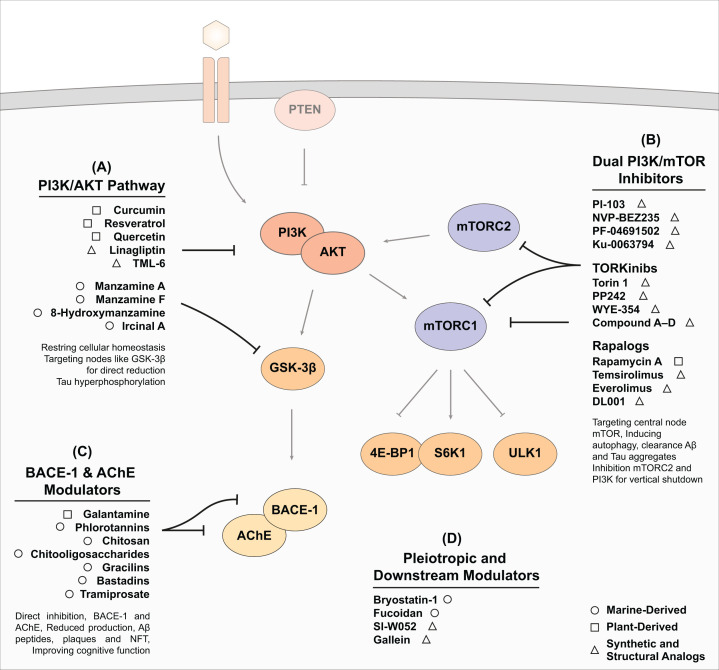
Schematic overview of mTOR inhibitor classes and their targets in AD To visualize the point of action of different classes of mTOR pathway modulators, the classes of (**A**) regulators of the PI3K/AKT axis and downstream target GSK-3β, (**B**) direct inhibitors of mTOR or dual PI3K/mTOR inhibitors, (**C**) modulators of BACE-1 and AChE, and (**D**) inhibitors with pleiotropic or downstream-target specific effects are shown along with targets and proposed outcomes of the modulation.

Considering the central role of mTOR signaling in AD, pharmacological modulation of the mTOR pathway holds strong therapeutic potential and warrants further investigation. In this review, we examine a diverse range of compounds with proposed mTOR-modulating activity, categorized by their origin, molecular targets, and mechanisms of action. First, we grouped the compounds into four mechanistic categories and visualized their interactions within the mTOR signaling network (Figure 2). The categories include (A) regulators of the PI3K/AKT axis, including inhibitors of GSK-3β and related kinases; (B) direct inhibitors of mTOR or dual PI3K/mTOR inhibitors; (C) modulators of BACE-1 and AChE as enzymes implicated in amyloid pathology and cholinergic dysfunction; and (D) compounds with partially characterized mechanisms, pleiotropic effects, or effector-related modulators.

**Figure 3:**
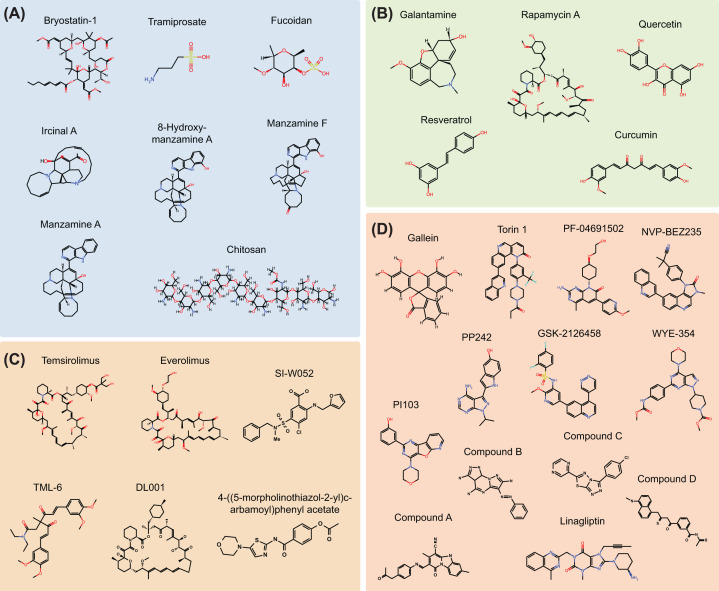
Chemical structures of mTOR signaling pathway modulators Chemical structures of modulators of the Alzheimer-related mTOR signaling pathway were obtained from PubChem or replicated based on the original reporting. The structures were grouped based on their origin as (**A**) marine-derived, (**B**) plant-based, (**C**) structural analogs, and (**D**) synthetic compounds.

In addition to classifications by mechanism of action, we grouped the 37 mTOR-modulating compounds based on their origin into the groups of marine-derived, plant-based or terrestrial, structural analogs, and synthetic compounds (Table 1) along with their chemical structures (Figure 3). While the classification does not necessarily reflect the mechanism of action or target specificity, it might facilitate understanding the source diversity of potential therapeutic agents modulating the mTOR pathway in neurodegenerative diseases and offer insight into pharmacokinetic properties, bioavailability, and safety profiles, which are essential for translational research.

**Table 1:** Overview of mTOR pathway-modulating compounds

Name	Source	Primary function	Additional and alternate functions	Reference
**Marine-derived compounds**				
Bryostatin-1	*Bugula neritina*	PKCε activation, synaptic plasticity, Aβ reduction (human subject studies)	Cytostatic; T-cell activation	[58,192,193]
Gracilins	*Spongionella gracilis*	BACE 1 inhibition; reduction of tau and ERK phosphorylation (*in vitro*)	Anti-inflammatory, mitochondrial protection	[58,194]
Manzamine A	*Acanthostrongylophora* sp.	GSK-3β & CDK-5 inhibition; reduces tau phosphorylation (*in vitro*/*in vivo*)	Cytotoxic, anti-HIV, antimalarial	[55,58]
Manzamine F	*Acanthostrongylophora* sp.	GSK-3β inhibition reduces tau hyperphosphorylation (*in vitro*/*in vivo*)	Antimalarial and antimicrobial activity (e.g. *Mycobacterium tuberculosis*)	[55,58]
8-Hydroxymanzamine A	*Acanthostrongylophora* sp.	GSK-3β inhibition reduces tau hyperphosphorylation (*in vitro*/*in vivo*)	Improved antimalarial activity	[55,58]
Ircinal A	*Acanthostrongylophora* sp.	Reduces tau hyperphosphorylation	Antimalarial and antimicrobial	[54,55]
Fucoidan	*Laminaria japonica, Fucus vesiculosus*	Caspase-3/9 inhibition; neuroprotection via mitochondrial pathways (*in vitro*/*in vivo*)	Antioxidant, anti-inflammatory, neuroprotective	[58,195,196]
Bastadine	*Ianthella basta*	BACE-1 inhibition (*in vitro*/*in vivo*)	Modulates Ca^2+^ membrane transport	[58]
Tramiprosate	Red seaweeds	Aβ aggregation inhibition	Anticonvulsant, hypothermic	[58,79]
Rapamycin A	*Streptomyces hygroscopicus*	Inhibition kinase activity mTORC1 (binding FKBP-12) (clinical trials)	Inhibiting carcinoma growth	[197]
Phlorotannins	*Ecklonia stolonifera*	Inhibition of AChE (*in vitro*/*in vivo*)	Anti-inflammatory effects via inhibition of pro-inflammatory cytokines and oxidative stress	[58,198]
Chitosan	Exoskeletons of crustaceans	Inhibits Aβ fibrillogenic; reduces oxidative stress & microglial inflammation & AChE activity (*in vitro*/*ex vivo*/*in vivo*)	Antioxidant, cholesterol binding; neuroprotective in PD models	[58,77,177]
Chitooligosaccharides	Chitosan oligomers	Inhibition of AChE; suppression of LPS-induced macrophage inflammatory responses via MAPK signaling pathway (*in vitro/in vivo*)	Antioxidant activity; cholesterol trapping	[77]
**Plant or terrestrial-derived inhibitors**				
Galantamine	*Galanthus nivalis*	AChE inhibition; increases cholinergic tone. Competitive inhibitor of AChE; neuroprotective activity (clinical trials)	Reduces oxidative stress, insulin resistance in MetS	[92,199–202]
Curcumin	*Curcuma longa*	Disrupts mTOR–Raptor; enhances autophagy (*in vitro*/*in vivo*)	Anticancer (proliferation inhibition) and apoptosis	[95,203]
Resveratrol	Polyphenolic flavonoid from grapes	Inhibits PI3K/Akt/mTOR pathway (*in vitro*/*in vivo*)	Anti-inflammatory; antioxidant; promotes cell death with rapamycin	[100,101,105]
Quercetin	Plant flavonoid	Activates PI3K/Akt; reverses neuronal LPS damage (*in vitro*/*in vivo*)	Anticancer; antiviral; anti-inflammatory	[108,204]
**Structural analogs and synthetic compounds**				
Temsirolimus	Synthetic rapamycin derivative	Inhibits mTORC1 (clinical trials); reduces tau and NFTs	Impairs protein synthesis	[117,205]
Everolimus	Synthetic rapamycin derivative	mTORC1/C2 inhibition; VEGF targeting	Treats renal carcinoma	[114,119]
DL001	Rapamycin analog	Strong mTORC1 selectivity (*in vitro*/*in vivo*)	Impairs glucose tolerance	[84]
SI-W052	Synthetic anthranilate analogue	Inhibits LPS-induced p-mTOR (*in vitro*/*in vivo*)	Anti-inflammatory (against IL-6)	[134]
TML-6	Synthetic curcumin analog	Inhibition APP and Aβ synthesis (*in vitro*)	Antioxidant activity	[132,133]
4-((5-morpholinothiazol-2-yl)carbamoyl)phenyl acetate	Nitazoxanide analog	Inhibits p70S6K and tau phosphorylation	Anti-parasitic	[135,206]
**Synthetic compounds**				
Gallein	Synthetic fluoran derivative	Inhibits Aβ-induced dendritic dystrophy and tau hyperphosphorylation (*in vitro/ex vivo*)	Inhibition of PPK1, PPK2A; metal indicator; anti-cancer activity	[139,207,208]
PI-103	Synthetic PI3K inhibitor	Inhibits PI3K/Akt/mTOR signaling pathway; neuroprotective effects (*in vitro*)	Anticancer activity (tumor growth inhibition); used in transplant research	[176,197,209]
NVP-BEZ235	Synthetic dual PI3K/mTOR inhibitor	Inhibits PI3K/Akt/mTOR pathway; reduces proliferation of tumor tissues (*in vitro*)	Anticancer activity; reduces PI3K phosphorylation	[197,209]
PP242	Synthetic mTOR inhibitor	Selective inhibition of mTORC1 and mTORC2 (*in vitro*)	Inhibition of vessel-like structure formation; anti-cancer activity	[209,210]
GSK-2126458	Synthetic dual PI3K/mTOR inhibitor	Potent inhibition of class I PI3K isoforms and mTORC1/2 (*in vitro*/*in vivo*)	Strong antiproliferative activity; induces apoptosis in tumors	[175,211]
Torin 1	Synthetic compound	mTOR kinase inhibitor (*in vitro*/*in vivo*)	Affects mRNA translation; anti-inflammatory, anti-cancer, and anti-aging effects	[209,212]
PF-04691502	Synthetic compound	Potential selective inhibitor PI3K and mTOR (*in vitro*)	Anti-cancer activity	[128,213]
WYE – 354	Synthetic compound	Competitive inhibitor (ATP) mTORC1/C2 (*in vitro*)	Anti-cancer activity	[124,125]
Linagliptin	Synthetic DPP-4 inhibitor; expressed in brain, pancreas, liver, and gut	Neuroprotective against Aβ-induced cytotoxicity and tau (*in vitro*/*ex vivo*/*in vivo* studies)	Alleviates symptoms of liver and inflammatory diseases; type 2 diabetes mellitus treatment	[214,215]
Compound A–D	Virtual screening (*in silico*)	ATP-competitive inhibitors of mTORC1 and mTORC2 (*in vitro*)	Anticancer effects: induces apoptosis and cell cycle arrest	[6]

Bioactive compounds were categorized by origin into marine-derived, plant-based, structural analogs, and synthetic compounds, summarizing the source, mechanism of action in AD, and additional biological effects.

### Marine-derived compounds

With its unique biodiversity, the marine environment is a rich source of structurally diverse and biochemically active compounds, many of which interact with fundamental cellular pathways, making them valuable for discovering novel modulators of signaling networks dysregulated in AD and neurodegeneration.

#### Bryostatin-1: protein kinase C modulation

Bryostatin-1 was isolated from the marine bryozoan *Bugula neritina* [46], initially identified due to its potent anti-neoplastic activity. Its primary mechanism of action is the modulation of protein kinase C (PKC), which is central to signal transduction and cell proliferation. Its activity was proven effective in preclinical models of numerous tumors, including lymphoma, melanoma, and ovarian carcinoma [47]. While the PKCε isoform is deficient in the brains of AD patients [48], activating the PKC pathway is shown to be neuroprotective [49]. The well-characterized ability to modulate PKC promotes non-amyloidogenic α-secretase processing of APP, resulting in a reduced production of toxic Aβ peptides [49,50]. Furthermore, with PKC as a downstream effector of mTORC2 signaling, bryostatin-1 was shown to influence the mTORC1 substrate p70 S6 kinase, suggesting a stabilization of the signaling network [51] and impairing tau-mediated microtubule disassembly [18]. While bryostatin-1 was reported to improve the cognition and activity of adults with moderate to severe AD [52] and can aid in restoring the blood-brain barrier (BBB) [53], the administration shows few side effects in the human body, making it an attractive therapeutic in AD treatment [46,47].

#### Manzamine alkaloids: GSK-3β inhibition

The manzamine alkaloids, isolated from marine sponges, were initially investigated for various bioactivities, including potent antimalarial, anti-inflammatory, and broad-spectrum kinase-inhibitory effects [54]. In neurodegenerative diseases, the therapeutic potential was highlighted in AD models as potent inhibitors of GSK-3β, with manzamine A shown to directly interfere with its activity, resulting in reduced tau hyperphosphorylation [55]. Its analogs, manzamine F and 8-hydroxymanzamine, share this GSK-3β inhibitory activity, with manzamine F exhibiting a more substantial antimalarial effect and 8-hydroxymanzamine further inhibiting CDK5 as an additional tau kinase [56]. Moreover, ircinal A, first isolated from a marine sponge (*Ircinia*), is a derivative of manzamine A, lacking a β-carboline group [57], and is believed to show a closely related function [55]. Manzamine A was reported to activate the PI3K/AKT pathway, indicating a direct link to mTOR activity [18,55], but also to promote autophagy [58], which are usually opposing functions. However, recent mechanistic profiling demonstrated that manzamine A potently induces autophagy by down-regulating RIP1, a key upstream regulator, thereby profoundly inhibiting the AKT/mTOR signaling pathway [59], highlighting its role as a dual-action agent by dampening mTOR activity while independently inhibiting GSK-3β.

#### Gracilins, phlorotannins, and bastadins: BACE-1 inhibition

As the primary driver of Aβ production, BACE-1 has proven resistant to multiple synthetic inhibitors, underscoring the value of identifying novel chemical scaffolds and studying natural BACE-1 inhibitors. Gracilins from the sponge *Spongionella gracilis* were first investigated for their diverse effects, including anti-inflammatory and immunosuppressive functions. When explored for neuroprotection, they were identified as direct inhibitors of BACE-1 and the ERK pathway. Gracilin A treatment significantly reduced the secretion of Aβ peptides from APP-overexpressing human neuronal cells [60]. However, further studies in animal models are critical to determine its therapeutic potential for AD.

Phlorotannins have a broad spectrum of known antioxidant and anti-cancer properties, including the induction of cell cycle arrest and apoptosis [61] and down-regulation of PI3K/AKT pathway signaling [62]. Furthermore, in AD research, they were subsequently identified as effective BACE-1 inhibitors [63], showing a broad range of potential applications in modulating AD progression.

In addition, bastadine alkaloids, first isolated from the marine sponge *Ianthella basta*, are proposed as direct inhibitors of BACE-1’s enzymatic activity [64]. However, the therapeutic development of bastadins has been limited, with further preclinical studies required to determine whether this enzymatic inhibition translates into a meaningful reduction in Aβ pathology.

#### Fucoidan, chitosan, and tramiprosate

The sulfated polysaccharide fucoidan was first isolated from marine invertebrates and brown seaweed, known for its anticoagulant, anti-inflammatory, and anticancer activities [65,66], with its antitumor activity ranging from apoptosis, inhibiting metastasis, enhancing the function of immune cells, and sensitizing cancer cells to conventional chemotherapies [67,68]. During the investigation of its broad biological effects, its neuroprotective potential was uncovered, activating the PI3K/AKT pathway while also inducing autophagy by activation of the AMPK pathway and downstream inhibitions of mTORC1, leading to neuroprotective effects against Aβ-induced neurotoxicity [69,70]. Moreover, fucoidan exhibits low oral bioavailability, with recent evidence confirming that its primary mechanism of action is indirect, operating via the microbiota-gut-brain axis (MGBA), repairing intestinal barrier integrity, and altering the colonic microbiome to produce neuroprotective short-chain fatty acids (SCFAs), which systematically dampen central neuroinflammation and downstream mTOR hyperactivation [71,72].

Chitosan, derived from chitin, is a well-established biomaterial in drug delivery and tissue engineering [73] but also possesses direct anti-tumor activity by inducing apoptosis and disrupting the mitochondrial membrane potential in cancer cells [74]. However, in AD, a different mechanism was identified, with chitosan inhibiting the mTORC1 downstream effectors p70S6K and 4EBP1, increasing immune responses, and preventing mTOR pathway-mediated tau hyperphosphorylation and microtubule disassembly [18,75]. While the biomedical applications of chitosan remain limited due to its poor solubility [76], its more soluble derivatives, chitooligosaccharides, act on additional targets, including BACE-1 and AChE, further reducing Aβ peptide levels and tau phosphorylation [77].

In addition, tramiprosate, a glycosaminoglycan mimetic originally identified in marine red algae, was investigated as a downstream effector of mTOR signaling [78]. Its primary mechanism of action involves binding specifically to soluble Aβ monomers, thereby stabilizing them in a non-fibrillar conformation and preventing their structural shift into toxic oligomers and fibrillar amyloid plaques [78]. While it demonstrated initial mechanistic promise in neutralizing Aβ-induced neurotoxicity, its clinical development faced severe translational hurdles due to unfavorable pharmacokinetic properties [79].

In summary, the marine environment offers a remarkable chemical diversity for modulating the mTOR network and related AD pathologies, ranging from specific kinase inhibitors, such as the manzamines, to multi-target agents like fucoidan, and includes compounds like Bryostatin-1 that have progressed to clinical evaluation. The overarching challenge remains translating the potent preclinical activity of these compounds into effective therapeutics by addressing challenges of bioavailability, BBB penetration, and clinical trial design. Ultimately, while these marine derivatives exemplify the pleiotropic advantages of natural products—simultaneously targeting kinases, aggregation, and neuroinflammation—their clinical viability hinges on overcoming their inherent pharmacokinetic limitations.

### Terrestrial and plant-derived compounds

While the marine environment offers a unique source of chemical diversity, the terrestrial world, including higher plants and soil-based microbes, has historically been the most prolific source of natural products for drug discovery.

#### Rapamycin: the foundational mTOR inhibitor

Already described in the 1970s as a metabolite of the soil bacterium *Streptomyces hygroscopicus*, rapamycin was initially investigated for its potent antifungal activity, leading to the discovery of its immunosuppressive and anti-proliferative effects on mammalian cells by forming a complex with FKBP12 and allosterically inhibiting mTORC1. Rapamycin was used in clinical practice to prevent organ transplant rejection and to reduce tumor cell growth by inducing G1 cell cycle arrest [80]. Its role as an mTORC1 inhibitor made rapamycin a research tool investigating preclinical AD models, since rapamycin-induced mTORC1 inhibition robustly promotes autophagy, leading to enhanced clearance of both aggregated Aβ and hyperphosphorylated tau [81–83]. While acute treatment inhibits mTORC1 with high specificity, prolonged exposure also disrupts mTORC2 assembly in some cell types [84]. However, the significant challenge of rapamycin’s systemic immunosuppression in an elderly population led to the development of rapalogs with modified properties to improve brain penetration and reduce peripheral side effects [84,85].

#### Galantamine: targeting the cholinergic system

Unlike most compounds in this review, galantamine was developed for AD, making it a valuable benchmark for plant-derived therapeutics. As a tertiary alkaloid isolated from plants of the Amaryllidaceae family (such as *Galanthus* spp.), galantamine shows a specific, competitive, and reversible inhibition of AChE [86–88], increasing the levels of acetylcholine in the brain and improving cognitive function in AD patients [88,89]. Clinically, this led to the approval of galantamine hydrobromide for mild to moderate AD, enhancing cholinergic function [87]. Moreover, confirming its safety profile in specific populations such as those with renal impairment [90], with further studies conducted to develop and validate alternative drug formulations, such as oral liquids, to ensure reliable bioavailability and improve ease of use for patients [91,92]. In addition to its primary action on AChE, some studies suggest the influence of galantamine on other signaling pathways relevant to AD pathology, including the allosteric modulation of nicotinic acetylcholine receptors (nAChRs) via JNK and AKT pathways, influencing autophagic processes related to Aβ peptide-induced apoptosis [18,93,94]. However, while directly linked to AD, even an indirect modulation of mTOR might not function as the primary therapeutic mechanism.

#### Polyphenols: curcumin, resveratrol, and quercetin

Curcumin is the principal polyphenolic compound in turmeric (*Curcuma longa*), extensively used in traditional medicine. Curcumin is a well-documented anti-cancer agent with pleiotropic effects, inhibiting proliferation and tumor growth, and inducing apoptosis [95]. Crucially, it is known to suppress the PI3K/AKT/mTOR pathway as a key mechanism in various cancers [4,95–97]. This established ability to modulate the mTOR axis provides a strong rationale for its use in AD, with the proposed mechanism involving inhibition of the insulin-like growth factor 1 (IGF-1) receptor as an upstream activator of the PI3K/AKT/mTOR pathway, resulting in reduced phosphorylation and activity of the mTORC1 substrate S6K1 [4,96,98] and pathological tau hyperphosphorylation [18].

Resveratrol is a natural stilbenoid polyphenol found in the skin of grapes, blueberries, and other fruits. In oncology, resveratrol is known to induce apoptosis and inhibit the proliferation of cancer cells, including acute lymphoblastic leukemia and multiple myeloma cells [99]. Recent studies highlight the anti-inflammatory and antioxidant effects [100,101]. The neuroprotective mechanism of resveratrol is primarily linked to its potent activation of the AMPK and SIRT1 pathways, which act as master regulators to inhibit mTORC1 [102,103]. Recent studies provide strong mechanistic evidence that this resveratrol-induced mTOR inhibition not only promotes the autophagic clearance of Aβ and tau aggregates but also profoundly mitigates microglial activation by suppressing the NF-κB inflammatory signaling network [101–107].

Quercetin is a prominent dietary flavonoid found in many fruits, vegetables, and grains, known for its potent antioxidant and anti-inflammatory properties. Its anti-cancer effects via the PI3K/AKT/mTOR signaling pathway are directly linked to the inhibition of key kinases, such as PI3K, with quercetin effectively reducing growth and survival signals driving tumor progression, leading to apoptosis and cell cycle arrest in cancer cells [108,109]. This mechanism is directly applicable to AD to reduce the pathological hyperactivated pathway in a neuroprotective manner and restore cellular homeostasis by promoting the autophagic clearance of Aβ and tau aggregates, while also reducing Aβ production by down-regulating mTOR’s influence on BACE-1 expression [110,111]. Furthermore, quercetin has been shown to reduce Aβ-induced toxicity through autophagy-dependent mechanisms, potentially involving pathways such as Abl depletion [112]. Like their marine counterparts, these plant-derived polyphenols underscore the immense value of broad-spectrum target engagement in a multifactorial disease such as AD. However, translating their potent in vitro multi-target efficacy into the clinic remains severely bottlenecked by their rapid metabolism and poor bioavailability.

### Structural analogs and synthetic compounds

While natural products are a vital source of bioactive scaffolds, they often exhibit suboptimal pharmaceutical properties, particularly in solubility, stability, and oral bioavailability. To address these issues, creating structural analogs by modifying the core pharmacophore of the natural product can enhance their drug-like characteristics. Covering the evolution of synthetic mTOR modulators, from direct analogs of natural products to novel molecules designed to target the pathway more precisely, researchers aim to overcome the pharmacokinetic hurdles of natural products. However, this pursuit of pharmacological precision often trades broad-spectrum benefits for narrow, highly specific inhibition, which can paradoxically trigger compensatory feedback loops [113].

#### Rapalogs: allosteric mTORC1 inhibitors

Temsirolimus is an ester of rapamycin specifically developed to improve its solubility for intravenous administration [114]. As the first mTOR-targeting drug approved by the FDA for the treatment of advanced renal cell carcinoma (RCC) [115,116], its mTORC1 inhibiting effects led to reduced cell cycle progression, limited tumor cell proliferation, and reduced angiogenesis by decreasing the production of vascular endothelial growth factor (VEGF) [115–117]. Specifically, temsirolimus has been shown to promote the autophagic clearance of Aβ in cellular and animal models, reducing apoptosis and improving memory abilities [118]. Although it shows improved solubility compared with rapamycin, its intravenous administration limits its use in AD and preclinical studies, as research generally focuses on orally available analogs.

Similarly, everolimus is a hydroxyethyl ether derivative of rapamycin, which significantly improves its metabolic stability and oral bioavailability compared with rapamycin and temsirolimus [114]. The mechanism of action relies on the formation of complexes with FKBP12, inhibiting mTORC1 activity. Due to its convenient oral administration, everolimus is approved for a wide range of cancers, including renal cell carcinoma, neuroendocrine tumors, and breast cancer [114,119] and shows high potential for long-term use in chronic diseases like AD. The therapeutic rationale is to leverage its mTORC1-inhibitory function to stimulate autophagy and clear neurotoxic proteins, with preclinical studies demonstrating that everolimus can reduce Aβ levels and improve memory deficits in mouse models of AD [120,121].

As an additional rapalog, DL001 was designed as a next-generation rapamycin analog for use in neurodegenerative disease, with increased binding affinity and specificity to mTORC1 and reduced off-target binding to mTORC2 to mitigate metabolic side effects. More specific and potent mTORC1 inhibition in AD might increase therapeutic benefits while minimizing side effects. By inhibition of the mTORC1 substrate S6K1, a proposed reduction in tau pathology and microtubule disassembly might prevent or treat tau-related aspects of AD [18,84].

#### ATP-competitive mTOR kinase inhibitors (TORKinibs)

While rapalogs show incomplete inhibition of mTORC1 and lack direct mTORC2 activity as limitations, a second generation of inhibitors directly targets the ATP-binding site within the kinase domain of mTOR (TORKinibs), potently inhibiting both mTORC1 and mTORC2. Torin 1 and PP242 are prime examples, developed with high potency and selectivity, with the complete inhibition of mTORC1 and mTORC2 activity leading to a more profound blockade of cancer cell proliferation and survival [122]. Moreover, PP242 was shown to be more effective than rapamycin at blocking cap-dependent translation, a key driver of tumor growth [123]. In AD, a more complete shutdown of the pathologically hyperactive mTOR pathway by inhibiting both complexes might induce autophagy more robustly, further clearing Aβ peptides and blocking the phosphorylation of tau via mTORC1/S6K1 or 4E-BP1 and potentially other mTORC2-related pathways implicated in tau pathology [18,123].

Similarly, WYE-354 and Ku-0063794 were developed as potent, specific, and ATP-competitive mTOR kinase inhibitors for cancer research [124,125]. Moreover, Ku-0063794 was reported to have no activity against other PI3K family kinases [126], making it a valuable compound for studying mTOR-specific effects [126]. With the compounds effectively suppressing cancer cell growth and inducing G1-phase cell-cycle arrest, the application in AD models is based on the same principle of potent dual inhibition of mTORC1 and mTORC2 and reducing phosphorylation of mTORC1 substrates like 4E-BP1 to modulate the progression of tau pathology and other mTOR-driven aspects of AD [125,126].

#### Dual PI3K/mTOR inhibitors

This third generation of inhibitors was developed for oncology to provide an even more profound blockade of the pathway by targeting both the upstream activators, such as PI3K and mTOR, at the central node, aiming to prevent feedback loops that lead to treatment resistance in cancer.

NVP-BEZ235 and PF-04691502 are well-characterized dual PI3K/mTOR inhibitors that entered clinical trials for various solid tumors [127,128]. By interfering with the entire signaling axis, which is highly effective at inhibiting tumor growth, proliferation, and survival [129], the strong connection between aberrant PI3K/AKT/mTOR signaling and AD pathology suggests that these dual inhibitors could be highly effective at inducing autophagy and reducing tau pathology more robustly. However, a higher risk of toxicity can be suggested, marking significant consideration for their use in chronic neurodegenerative disease.

Moreover, PI-103 was among the first compounds to exhibit potent dual inhibition of PI3K and mTOR, demonstrating strong anti-leukemic activity in preclinical models, validating the dual-inhibition strategy for cancer treatment [130]. As a well-characterized dual PI3K/mTOR inhibitor, it has a rationale for use in AD, offering comprehensive inhibition of the hyperactive pathway, similar to NVP-BEZ235 and PF-04691502.

#### In silico-generated, structural analogs, and other synthetic modulators

Beyond traditional screening of existing chemical libraries, modern drug discovery increasingly leverages computational methods to rationally identify novel chemical scaffolds. Employing a virtual screening of compounds for their predicted binding affinity to the kinase domain of mTOR, Wang et al. identified 41 novel compounds. Subsequent *in vitro* testing was conducted to confirm the inhibitory activity, demonstrating a selectivity for mTORC1 and mTORC2 [6]. While the specific mechanism of action of selective mTOR inhibition remains unknown, this provides a robust proof of concept for the development of new mTOR modulators.

While curcumin shows potent anti-cancer and anti-inflammatory activity, its clinical development has been severely hampered by its poor stability, rapid metabolism, and low bioavailability [131]. TML-6 is a synthetic monocarbonyl analog of curcumin, designed to overcome these limitations while retaining the therapeutic activity. The rationale for using TML-6 is to enhance curcumin-related neuroprotective effects, with preclinical studies showing a more stable and potent activity by suppressing mTOR phosphorylation, thereby reducing Aβ deposition and accumulation, and limiting disease progression [132,133].

SI-W052 is a novel synthetic anthranilate analog, identified for its potent anti-neuroinflammatory effects. In AD mouse models, SI-W052 exerts anti-inflammatory effects, improves cognitive function, and reduces phosphorylated mTOR levels, thereby promoting autophagy and enhancing Aβ clearance. However, the direct molecular target of SI-W052 that leads to mTOR inhibition remains under investigation [134].

The morpholinothiazole compound 4-((5-morpholinothiazol-2-yl)carbamoyl)phenylacetate was identified through high-throughput screening for inhibitors of the PI3K/AKT/mTOR pathway for anticancer applications. By inhibiting the phosphorylation of p70S6K as a substrate of mTORC1, downstream hyperphosphorylation of tau, and subsequent microtubule disassembly, the compound can be suggested as a potent inhibitor of the mTOR signaling pathway in AD, by reducing the phosphorylation of tau [135].

As an FDA-approved drug for treating type 2 diabetes, the primary mechanism of action of linagliptin is inhibition of dipeptidyl peptidase 4 (DPP-4), an enzyme that degrades incretin hormones such as GLP-1. By inhibiting DPP-4, linagliptin increases GLP-1 levels, improving insulin secretion and glucose control [136]. Given the strong link between diabetes and dementia, enhancing the neuroprotective GLP-1 signaling system by linagliptin can reduce neuronal death and neuroinflammation [137]. It is proposed that these neuroprotective effects are mediated through an indirect modulation of the AKT/mTOR signaling pathway [138].

Moreover, gallein is a small organic molecule identified as an inhibitor of G protein βγ-subunit signaling. Its potential relevance to AD was identified by its influence on the mTOR signaling pathway, specifically inhibiting the phosphorylation of the mTORC1 substrate S6K1, which suggests that gallein can interfere with the hyperphosphorylation of tau and subsequent microtubular disassembly, making it a potential tool for mitigating tau pathology [139].

## Translational challenges and clinical perspectives

### Chemical diversity and pharmacological precision

While the variety of mTOR modulators can be categorized into natural products and synthetic compounds, this further represents two distinct approaches to utilizing the inherent complexity and pleiotropy of nature or the precision and optimized pharmacology of rational design. Natural products, sourced from unique and biodiverse marine and terrestrial environments, offer structural novelty and often exhibit pleiotropy. With AD driven by a complex interplay of protein aggregation, neuroinflammation, oxidative stress, and metabolic dysregulation, this enables the simultaneous engagement of multiple cellular pathways. Plant-derived polyphenols, such as curcumin and resveratrol, offer therapeutic potential beyond their influence on the PI3K/AKT/mTOR axis. Acting as potent antioxidants and anti-inflammatory agents, simultaneously addressing inflammation, oxidative stress, and proteostasis may be more effective than narrowly focused, single-target therapies for rebalancing the dysregulated system [105,140]. However, this broad-spectrum activity presents significant challenges, as many natural products have suboptimal pharmacokinetic profiles that severely limit their clinical application [141]. Despite potent preclinical activity, the translation of curcumin has been hindered primarily by poor bioavailability and rapid metabolism [142]. Moreover, fucoidan exhibits similarly low oral bioavailability, with emerging evidence suggesting a strong indirect effect mediated by modulation of the gut microbiome [143]. Furthermore, the mechanisms of action of natural products are often poorly defined, creating additional challenges for rational drug development. While manzamine A was initially reported to inhibit GSK-3β, its complete mechanism appears more complex, involving multiple cellular pathways that are still being elucidated [144]. However, natural products remain an invaluable and irreplaceable resource, providing novel chemical scaffolds, such as the PKC modulator bryostatin-1 or the BACE-1-inhibiting gracilins, which are structurally distinct from those typically found in synthetic libraries and can serve as inspiration for the development of more drug-like synthetic analogs [145,146]. Moreover, the perceived safety of many natural products, often stemming from their long history of use in traditional medicine or their presence in the human diet, can facilitate pathways to clinical approval and improve patient acceptance [147].

In contrast, rational drug design and synthetic chemistry systematically address the specific limitations of natural compounds by improving their pharmacological properties and elucidating their mechanisms of action. Due to the poor bioavailability of rapamycin, the synthetic rapalogs everolimus and temsirolimus were specifically designed to significantly enhance their metabolic stability and oral availability for chronic use [148,149] or improve solubility for intravenous administration [115,116], respectively. Similarly, to address the poor stability and rapid metabolism of curcumin, synthetic monocarbonyl analogs, such as TML-6, were developed to retain therapeutic activity in a more drug-like form [132,142,150]. Furthermore, the mechanistic clarity of synthetic inhibitors enables an iterative development process that is impossible with poorly defined natural products. While first-generation rapalogs were found to be limited by an AKT-activating feedback loop, second-generation ATP-competitive inhibitors (TORKinibs), inhibiting both mTORC1 and mTORC2, and third-generation dual PI3K/mTOR inhibitors were created to provide an even more comprehensive vertical blockade of the signaling cascade [151–153], highlighting the distinct advantage of synthetic chemistry in creating potent, precise, and pharmacologically optimized therapeutic agents to overcome known mechanisms of resistance. However, the increased precision and targeting a single node in a complex disease network can be ineffective, as demonstrated by the clinical failures of many highly specific synthetic BACE-1 inhibitors, whereas natural inhibitors like gracilins may offer broader, more beneficial effects [60,154,155].

### mTOR modulation: a double-edged sword

While hyperactivation of the mTOR pathway is implicated in AD pathology, any therapeutic approach must navigate the fine line between dampening pathological signaling and preserving essential physiological functions to achieve neuroprotective benefits without systemic toxicity [30]. The extensive clinical use of mTOR modulators in oncology and organ transplantation highlights side effects, such as high-dose applications of rapamycin and its analogs like everolimus and temsirolimus, which are effective at controlling cell proliferation, but are additionally associated with a dose-limiting, significant burden of adverse events [156], including metabolic and dermatological toxicities, as well as systemic effects like immunosuppression [157]. Unlike in oncology, a long-term, chronic treatment for an elderly population might further promote metabolic complications, such as diabetes or increased glucose intolerance, given the high prevalence of comorbidities like insulin resistance and cardiovascular disease [158], with additional immunosuppressive effects of mTOR inhibitors [159,160], further raising serious concerns for an aging population already susceptible to infections. However, emerging evidence suggests a navigable therapeutic window, with studies in aging mice demonstrating that intermittent or low-dose rapamycin application can extend lifespan and confer health benefits while mitigating some of the adverse metabolic effects seen with chronic, high-dose treatment [161]. Moreover, a clinical trial demonstrated that low-dose everolimus (RAD001) was relatively well-tolerated and improved immune function by enhancing the response to influenza vaccination [162]. Crucially, transitioning these compounds from oncology to neurology requires a paradigm shift in dosing and delivery. While cancer protocols often utilize maximum tolerated doses delivered intravenously (as with temsirolimus) to aggressively arrest tumor growth, AD treatment necessitates chronic, low-dose, oral, or targeted central administration (such as Everolimus) to achieve neuroprotective autophagy without triggering systemic immunosuppression or metabolic toxicity. The potential for fine-tuning the mTOR pathway to dampen its activity and achieve a neuroprotective effect suggests repurposing these agents for AD, with the goal to identify dosing strategies that can decouple the desired central nervous system (CNS) benefits from the deleterious peripheral side effects.

Since clearing pathogenic protein aggregates, such as Aβ and tau, is the primary therapeutic target in AD, inducing autophagy through selective inhibition of mTORC1 is desirable [163]. However, prolonged, chronic exposure to rapamycin can disrupt the assembly and function of mTORC2 in certain tissues, a process that is thought to underlie many of the drug’s adverse metabolic effects, such as glucose intolerance and insulin resistance [164]. While ATP-competitive rapalogs, such as Torin 1 and PP242, provide a more complete blockade of mTOR signaling [165], the inhibition of mTORC2, and the consequent impairment of AKT signaling and insulin sensitivity, could be detrimental in the context of AD, where brain insulin resistance is a known pathological feature [164]. With recent evidence from AD mouse models of protective functions of mTOR activation in microglia, leading to improved clearance of Aβ plaques and better cognitive outcomes [166] and contrary evidence of increased Aβ plaque burden in response to systemic treatment with rapamycin [167], cell-type-specific mTOR modulation [168] might dampen neuronal mTORC1 activity while preserving or even enhancing microglial mTOR function.

Additionally, the strategic decision of where to intervene in the PI3K/AKT/mTOR signaling cascade has significant consequences for both efficacy and off-target effects, with rapalogs and TOR kinase inhibitors (TORKinibs) directly targeting mTOR as the key convergence point for numerous upstream pathological signals. However, feedback mechanisms within the pathway exhibit fundamental limitations, as the inhibition of mTORC1 phosphorylates and degrades insulin receptor substrate-1 (IRS-1) [169], leading to hyperactivation of upstream PI3K/AKT signaling and counteracting the intended therapeutic effects [152]. While dual PI3K/mTOR inhibitors, such as NVP-BEZ235 and PI-103, were developed to target additional upstream activators and prevent the feedback activation of AKT [153,170], significant toxicity was observed due to the general inhibition of class I PI3K isoforms and mTOR complexes [171]. Moreover, targeting specific downstream effectors, including S6K1, might be a more refined approach to achieve a therapeutic outcome with greater precision, as S6K1 expression is up-regulated in the brains of AD patients [172,173], and might shift the balance of efficacy and safety for a chronic neurodegenerative disease [174].

### Quantitative comparisons and preclinical limitations

The comparative analysis of the compounds, based on their origins and mechanistic targets, reveals fundamental trade-offs between structural novelty and pharmacological precision (Table 2). Structural novelty and pleiotropic effects demonstrated by natural products are significant advantages, as AD is driven by a complex interplay of protein aggregation, oxidative stress, and inflammation; the ability of plant and marine derivatives to engage multiple cellular pathways simultaneously offers broad systems-level correction. For example, marine-derived manzamine A acts as a highly specific dual inhibitor, targeting GSK-3β with an IC_50_ of 10.2 μM and CDK5 with an IC_50_ of 1.5 μM [55]. Clinically, intravenous bryostatin-1 at 25 μg/m^2^ has demonstrated significant preservation of cognitive function in moderately severe AD cohorts (+1.83 MMSE score improvement at 3 h). However, these broad-spectrum benefits are heavily counterbalanced by suboptimal pharmacokinetic profiles.

**Table 2:** Overview of experimental models and reported quantitative efficacy of select mTOR modulators

Compound	Class and origin	Assay, context, experimental model, and quantitative efficacy	Reference
Bryostatin-1	Pleiotropic, Marine	**Clinical Trials** Phase IIa and expanded access trials in human adults with moderate-to-severe AD (baseline MMSE 10–14), +1.83 MMSE score at 3 h, Peak PKCε activation under 1 h (25 μg/m^2^, IV)	[50]
Fucoidan	Pleiotropic, Marine	***In silico*** molecular docking simulations for PI3K (PI3K binding free energy = −9.64 kcal/mol)	[143]
Tramiprosate	Pleiotropic, Marine	**Clinical** APOLLOE4 Phase III trial mild-to-moderate AD (baseline MMSE scores of 16–26) (52% nominally significant slowing of cognitive decline (ADAS-Cog13) and a 26% reduced rate of hippocampal atrophy, 100 mg or 150 mg twice daily (BID))	[79]
Manzamine A	PI3K/AKT Pathway, Marine	***In vitro*** enzymatic assays (GSK3 inhibition 73.2% at 25 μM; GSK-3β IC_50_ = 10.2 μM; CDK5 IC_50_ = 1.5 μM), SH-SY5Y human neuroblastoma cells (decreases tau phosphorylation at 5 μM), BBB modeling MDR-MDCK monolayers (demonstrates BBB penetrance)	[55,216,217]
Manzamine F	PI3K/AKT Pathway, Marine	***In vitro*** enzymatic assays (GSK3 inhibition 29.9% at 25 μM; GSK-3β IC_50_ = 16.6–25 μM)	[217]
8-Hydroxymanzamine	PI3K/AKT Pathway, Marine	***In vitro*** enzymatic assays (GSK3 inhibition 86.7% at 25 μM; GSK-3β IC_50_ = 4.8 μM)	[217]
Ircinal A	PI3K/AKT Pathway, Marine	***In vitro*** enzymatic assays (Inactive directly on GSK-3β binding), cell-based SH-SY5Y assays (moderately reduce tau hyperphosphorylation)	[216,217]
Bastadines	BACE-1/AChE Modulator, Marine	***In vitro*** BACE-1 enzymatic cleavage assay (IC_50_ = 0.3 μM), APP-overexpressing cells (IC_50_ = 2.8 μM)	[218]
Phlorotannins	BACE-1/AChE Modulator, Marine	***In vitro*** enzymatic assays 8,8′-bieckol (AChE IC_50_ = 4.59 μM; BACE-1 IC_50_ = 1.62 μM)	[219]
Chitosan and chitooligosaccharides	BACE-1/AChE Modulator, Marine	***In vitro*** AChE enzymatic assay (AChE IC_50_ = 9.2 to 56.5 μg/ml), PC12 pheochromocytoma cells (disrupts self-assembly of Aβ peptides)	[7,8]
Galantamine	BACE-1 and AChE Modulator, Plant	***In vitro*** AChE enzymatic assays (AChE IC_50_ = 0.99 μM), Clinical Mild-to-moderate AD human cohorts (reduces oxidative stress, insulin resistance)	[87]
Resveratrol	PI3K/AKT Pathway, Plant	***In vitro*** BACE-1 enzymatic fluorescence assays (BACE-1 IC_50_ = 14.89 μM)	[100]
Rapamycin	Microbial	***In vitro*** enzymatic assays (mTORC1 IC_50_ ∼ 0.1 nM; mTORC2 insensitive); **Clinical** CARPE DIEM (Phase 1), REACH (Phase 2), and ERAP (Phase IIa) trials in MCI/Early AD (evaluated clinically at 1 mg/day or 7 mg/week)	[84,178]
Everolimus	Rapalog, Synthetic	***In vitro*** A549 human lung carcinoma cells (proliferation IC_50_ = 1.0 nM); ***In vivo*** murine AD models, intrathecal administration, and pharmacokinetic mouse models (mTOR EC_50_ = 6 nM; Brain-to-Plasma ratio = 3:1 at 30 mg/kg IV)	[120]
Temsirolimus	Rapalog, Synthetic	***In vitro*** assays (Direct mTOR kinase IC_50_ = 1.76 μM, FKBP12 independent)	[51,117]
DL001	Rapalog, Synthetic	***In vitro*** targeted mTORC1/mTORC2 kinase binding assays (Selective mTORC1 IC_50_ = 74.9 pM)	[84]
Torin 1	TORKinib, Synthetic	***In vitro*** kinase panels (mTORC1 IC_50_ = 2 nM, mTORC2 IC_50_ = 10 nM)	[220]
PP242	TORKinib, Synthetic	***In vitro*** enzymatic kinase panels (mTOR and PKCα) (Dual mTORC1/mTORC2 IC_50_ = 8 nM; PKCα IC_50_ = 49 nM); ***In vivo*** LS174T colon cancer xenograft mouse model (inhibition of vessel-like structure formation)	[123]
WYE-354	TORKinib, Synthetic	***In vitro*** DELFIA kinase assay, HEK293 cells (mTOR IC_50_ = 4.3–5.0 nM, PI3K selectivity)	[221]
PI-103	Dual Inhibitor, Synthetic	***In vitro*** kinase panels (PI3Kα IC50 = 8 nM, mTORC1 IC_50_ = 20 nM, mTORC2, IC_50_ = 83 nM, DNA-PK IC_50_ = 2 nM)	[222,223]
NVP-BEZ235	Dual Inhibitor, Synthetic	***In vitro*** enzymatic kinase panels (mTOR IC_50_ = 20.7 nM; PI3K IC_50_ = 4 nM); ***In vivo*** T41 transgenic AD mouse model and pharmacokinetic models (Brain-to-Plasma ratio = 1.7; Reduced tumor size and weight, suppression of energy metabolism)	[129,224]
PF-04691502	Dual Inhibitor, Synthetic	***In vitro*** kinase panels (PI3Kα Ki = 1.8 nM; mTOR Ki = 16 nM), U87MG glioblastoma lines (Proliferation IC_50_ = 179 nM)	[225]
GSK2126458	Dual Inhibitor, Synthetic	***In vitro*** enzymatic PI3K/mTOR kinase inhibition panels (mTORC1 IC_50_ = 0.18 nM; PI3K IC_50_ = 0.019 nM)	[175]
Quercetin	PI3K/AKT Pathway, Plant	***In vitro*** cytostatic assays in C4-2B/22Rv1 models (Cytostatic IC_50_ = 21.54 to 24.37 μM), hepatic stellate cells (Cytostatic IC_50_ = 27.2 μM)	[226,227]
Curcumin	PI3K/AKT Pathway, Plant	***In vitro*** enzymatic/cellular assays (mTOR inhibitor IC_50_ ∼ 20 to 50 μM)	[228]
TML-6	PI3K/AKT Pathway, Synthetic	***In vitro*** Huh7 cells (Reduces p-mTOR by 30% at <3.93 μg/ml), N2a APPswe models (Suppresses Aβ40 and Aβ42 production at 2.09 μg/ml)	[132]
SI-W052	Pleiotropic, Synthetic	***In vitro*** microglial models SIM-A9/LPS-stimulated (Inhibits LPS-induced p-mTOR, suppresses IL-6, TNF-α)	[134]
Gallein	Pleiotropic, Synthetic	***In vitro*** primary hippocampal neurons (5 μM blocks APP-T668 phosphorylation and BACE-1 binding); ***In vivo*** 3×Tg-AD murine models (mitigates overt synapse loss and blocks abnormal tau phosphorylation)	[229,230]

For example, curcumin exhibits notoriously poor aqueous solubility and rapid metabolism, which severely restricts its clinical application. To overcome this, synthetic analogs such as TML-6 were developed to prevent rapid degradation and activate neuroprotective pathways with 6.6 to 12.1 times the potency of the natural compound [132,140]. Additionally, their exact mechanisms are often complex and evaluated using disparate metrics, such as resveratrol’s modest BACE1 IC_50_ of 14.89 μM or galantamine’s AChE IC_50_ of 0.99 μM, making direct potency comparisons to synthetic kinase inhibitors difficult [87,100].

In addition, while unmodified rapamycin exhibits potent subnanomolar affinity for mTORC1 (IC50 0.1 nM), its clinical utility is hampered by unpredictable oral bioavailability and poor aqueous solubility. To systematically address these limitations, rapalogs were synthetically developed to optimize metabolic stability and oral bioavailability for chronic use [148] or to improve solubility for intravenous administration [117]. Furthermore, synthetic inhibitors offer high mechanistic clarity and vastly superior quantitative potency, with Temsirolimus and Everolimus achieving specific mTORC1 inhibition at EC_50_ values of 56 nM and 6 nM, respectively. Moreover, the engineered rapalog DL001 exhibits highly selective mTORC1 inhibition with an IC_50_ of 74.9 pM [117,120,148]. However, by binding FKBP12 rather than the kinase active site, they fail to fully suppress key mTORC1 substrates, including 4E-BP1, and completely spare mTORC2. Furthermore, this blunt inhibition of mTORC1 degrades IRS-1, triggering compensatory feedback loops that hyperactivate upstream PI3K/AKT signaling and counteract therapeutic efficacy [152]. To overcome the biochemical limitations of rapamycin and its analogs, newer generations of inhibitors were designed to achieve a vertical blockade of the hyperactive PI3K/AKT/mTOR signaling cascade and to fully suppress targets that are natively resistant to rapamycin. These compounds demonstrate profound quantitative potency. For instance, TORKinib PP242 aggressively inhibits both mTORC1 and mTORC2 with an IC_50_ of 8 nM [123]. Similarly, the dual-inhibitor NVP-BEZ235 suppresses mTOR at 20.7 nM and PI3K between 4 and 75 nM, while GSK2126458 exhibits extraordinary sub-nanomolar potency of mTORC1 (IC_50_ = 0.18 nM) and PI3K (IC_50_ = 0.019 nM) [175]. However, the increased potency and comprehensive inhibition lead to an even higher risk of generalized toxicity [176], restricting essential physiological functions, creating a narrow therapeutic window for a chronic neurodegenerative disease, and often restricting maximum tolerated *in vivo* doses.

However, a major challenge in the preclinical evaluation of mTOR modulators for AD is the absence of standardized *in vitro* and *in vivo* models. Currently, heterogeneous experimental contexts are utilized, ranging from APP-overexpressing human neuronal cells for evaluating gracilin A [60] to Aβ-stimulated PC12 cells for chitooligosaccharides [177]. This variation, alongside diverse transgenic mouse models with distinct timelines and pathologies, severely limits direct quantitative comparisons of compound potency, as reported efficacy metrics depend heavily on the specific models and dosing routes employed. While compounds like SI-W052 demonstrate systemic anti-inflammatory effects in AD mouse models [134], the rapalog everolimus required direct central administration in preclinical studies to bypass the BBB and elicit therapeutic reductions in Aβ levels [120].

### The clinical landscape

The clinical translation of mTOR inhibitors for AD remains limited, with initial trials highlighting challenges and advancing understanding of this approach’s feasibility. The Phase 1 open-label pilot study CARPE DIEM (NCT04200911) investigated patients with mild cognitive impairment or early AD treated with daily low-dose rapamycin. While the drug was not detectable in the CNS, indicating a failure to cross the BBB at a clinically manageable oral dose [178], additional paradoxical effects on AD and inflammatory biomarkers underscore the complex biological effects and the need for careful monitoring [178]. Moreover, the Phase 2 REACH clinical trial (NCT04629495) is a larger, 12-month, placebo-controlled study evaluating the daily oral administration of rapamycin (1 mg) and includes more comprehensive endpoints, including PET, MRI, and CSF analysis, to assess target engagement and downstream effects. In contrast, the ERAP trial (NCT06022068) is testing a higher weekly dose of rapamycin (7 mg), with the primary endpoint being a change in cerebral glucose uptake measured by FDG-PET as a direct test of a physiological effect in the brain [179]. The outcomes of these biomarker-driven trials might determine if there is a viable path forward for rapamycin itself. Meanwhile, despite extensive use in oncology [180], no major active AD trials of the rapalog everolimus have been conducted, likely because preclinical data showed poor BBB penetration, which is necessary for direct central administration [120].

While early trials of direct mTOR-modulating compounds highlighted the difficulty of crossing the BBB, the downstream modulator tramiprosate failed to meet primary cognitive endpoints in Phase III clinical trials in patients with mild-to-moderate AD [79]. Retrospective analyses reveal that this clinical failure was primarily driven by translational challenges, including high inter-individual pharmacokinetic variability, inconsistent CNS bioavailability, gastrointestinal intolerance, and the delayed targeting of Aβ aggregation in moderate AD stages [181]. However, recent clinical data confirm that ALZ-801 (valiltramiprosate; NCT04693520; APOLLOE4, NCT04770220), an optimized prodrug of tramiprosate, significantly stabilizes systemic bioavailability, reduces gastrointestinal side effects, and decreases hippocampal atrophy in targeted APOE4-carrier populations [182,183], highlighting the potential of natural product derivatives as effective clinical AD therapeutics using early-stage pharmacokinetic optimization and biomarker-driven patient stratification.

## Future directions and conclusion

### Gaps in knowledge and delivery strategies

While the rationale for repurposing mTOR modulators for AD is strong, significant challenges are limiting clinical translation. A primary concern of translating therapeutics for the CNS is the BBB. Despite promising preclinical results in animal models, clinical translation has failed to achieve therapeutic concentrations in the human brain, as demonstrated in clinical trials of oral rapamycin in patients with mild cognitive impairment [178]. Similarly, the rapalog everolimus exhibits poor CNS penetration, with preclinical studies requiring direct central administration to elicit therapeutic effects [120]. While increasing the systemic dose would lead to intolerable peripheral toxicity [158], encapsulation of mTOR pathway modulators using nanoparticles (NPs), such as nanostructured lipid carriers or extracellular vesicles [184], might evade clearance and increase receptor-mediated transcytosis through the BBB endothelium to actively transport the drug into the CNS [185,186]. Moreover, recent insights have been gained using an FKBP12-dependent brain-permeable mTOR inhibitor (RapaLink-1) administered in conjunction with a brain-impermeable FKBP12 ligand (RapaBlock) to achieve systemic neutralization and CNS-exclusive therapeutic effects [187], widening the therapeutic window by engineering tissue selectivity as a priority for future research to overcome the most critical barrier to clinical translation.

Moreover, to advance mTOR modulators for AD, a robust and translatable biomarker panel is essential to observe target engagement and biological effects [188], as demonstrated by the initial rapamycin trial showing undetectable drug levels in the CNS [178]. Hyperactivation of the PI3K/AKT/mTOR pathway observed in AD might be heterogeneous among patients [18], further highlighting the need for biomarkers in patient stratification to identify patients likely to benefit from mTOR pathway modulation and manage the known metabolic side effects of these drugs [158].

The inherent complexity of AD pathogenesis and limitations of monotherapies underscore the need for multi-target, combinational approaches. To overcome limitations in autophagy induction, simultaneous activation of mTOR-independent pathways could produce a more robust and sustained autophagic response [189], as demonstrated previously using trehalose [190]. Moreover, therapeutic approaches on the mTOR signaling pathway might be complemented by direct targeting of tau or Aβ to enhance clearance capacity via mTOR inhibition and induction of autophagy [191].

### Conclusion

Repurposing mTOR modulators from oncology is a compelling, rationale-driven strategy for AD, targeting a shared hallmark of pathological pathway hyperactivation. While existing modulators are broadly derived from two distinct approaches of harnessing the chemical diversity of nature and applying the pharmacological precision of synthetic chemistry, integration of both offers a powerful path forward. Natural products, sourced from unique terrestrial and marine environments, provide an invaluable reservoir of novel chemical scaffolds and pleiotropic effects. This multi-target activity can be advantageous in complex, multifactorial diseases like AD. However, their clinical translation is frequently stalled by poor pharmacological properties, such as low bioavailability and an inability to cross the BBB. In contrast, synthetic compounds are born from rational design, created to overcome these limitations, offering enhanced stability, specificity, and potency. However, highly specific inhibitors can trigger compensatory feedback loops, and their narrow focus may be inadequate for the brain's complex, cell-specific environment, where mTOR’s role can be protective in some cells and pathological in others. The proposed future of this therapeutic strategy relies on the synergy between these two approaches, using natural compounds as the blueprint for novel mechanisms, while synthetic chemistry provides the tools to build next-generation therapeutics to “fine-tune” the mTOR network, beyond blunt inhibition toward more sophisticated, cell-type-specific agents. Combining nature’s ingenuity with synthetic precision makes the continued pursuit of mTOR modulation a promising frontier in the search for a meaningful AD therapy.

## Abbreviation

4E-BP1: Eukaryotic initiation factor 4E-binding protein 1
AChE: Acetylcholinesterase
AD: Alzheimer’s disease
APP: Amyloid precursor protein
Aβ: Amyloid-beta
BACE-1: β-site amyloid precursor protein cleaving enzyme 1
BBB: Blood-brain barrier
CNS: Central nervous system
DPP-4: Dipeptidyl peptidase 4
GSK-3β: Glycogen synthase kinase-3β
IGF-1: Insulin-like growth factor 1
IRS-1: Insulin Receptor Substrate-1
MetS: Metabolic syndrome
mTOR: Mammalian target of rapamycin
nAChRs: Nicotinic acetylcholine receptors
NFTs: Neurofibrillary Tangles
PHFs: Paired helical filaments
PIKK: Phosphatidylinositol 3-kinase-related kinase
PKC: Protein Kinase C
RCC: Renal cell carcinoma
RTKs: Receptor tyrosine kinases
S6K1: Ribosomal protein S6 kinase
VEGF: Vascular Endothelial Growth Factor
